# Separating Sampling Bias From Abundance Shows That Different Methods Catch Different Wild Bees

**DOI:** 10.1002/ece3.73060

**Published:** 2026-02-12

**Authors:** Max W. McCarthy, Dylan T. Simpson, Andrew H. Aldercotte, Colleen Smith, Tina Harrison, Rachael Winfree

**Affiliations:** ^1^ Department of Ecology, Evolution, and Natural Resources Rutgers University New Brunswick New Jersey USA; ^2^ Chicago Botanic Garden Glencoe Illinois USA

**Keywords:** bias, community ecology, pollinators, sampling, wild bees

## Abstract

1. Ecological community sampling methods have taxonomic biases, producing samples where relative abundances of taxa may differ from the underlying sampled community. Evaluating sampling methods' relative biases is therefore necessary for accurately interpreting community data. Wild bees (Hymenoptera: Apoidea) have been the focus of intensive community sampling and many studies have compared the properties of samples collected by different methods. However, comparative studies have often conflated differences in sampling bias with differences in effort and absolute abundance between methods, potentially obscuring methods' true biases.
2. Here, we compare wild bee communities in the northeastern United States as sampled by pan traps, vane traps, and hand netting. Using a dataset of simultaneous sampling by different methods, we compare sample richness and composition between pairs of methods while accounting for differences in the overall number of bees sampled by each.
3. For a given number of individuals sampled, hand netting captured more bee species than pan traps, which captured more species than vane traps. Pan traps sampled a different pool of species than either of the other two methods. Of 21 bee genera analyzed, 8 were overrepresented in pan trap samples relative to hand netting, whereas 7 were relatively underrepresented in pan traps. When compared against vane traps, 4 genera of 20 were relatively overrepresented in pan traps, whereas 6 were relatively underrepresented. Pan traps poorly represented very large‐bodied genera as compared with the other methods.
4. We find pervasive biases in bee community sampling methods, with most genera showing significant differences in relative abundance in at least one methodological comparison. At times, genera were relatively underrepresented even by methods that collected them in higher absolute abundance. Since bias is unavoidable in community sampling, studies must measure taxon‐specific biases in the context of their system and evaluate the robustness of analytical results.

## Graphical abstract

Comparing community sampling methods' relative taxonomic biases is critical to interpreting the data they collect, but measuring bias explicitly is difficult when methods also produce different sample sizes. Here, we control for absolute abundance while comparing sample composition and richness of three common methods for sampling wild bee communities. We find that methods show varied biases for different genera and size classes of bees, with several common taxa among those most sensitive to choice of sampling method.

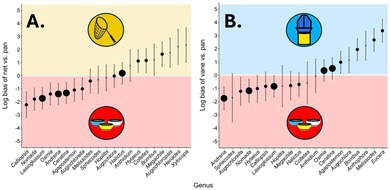

## 1 Introduction

Taxonomic sampling bias—the tendency of sampling methods to detect organisms in numbers disproportionate to their true abundances—is a persistent difficulty when surveying ecological communities (Elphick 2008). Community samples exhibit bias because sampling methods tend to favor species that exhibit specific behaviors (e.g., attraction to a certain visual or chemical cue) or have physical traits that increase their relative detectability (Biro 2013; Engel et al. 2017). Interpreting community data without knowing the biases of methods used to gather it can in turn lead to incorrect conclusions regarding population abundances, community assembly, species interactions, and other key ecological processes (Elphick 2008; Rhoades et al. 2017; Lee and Guénard 2019; Tronstad et al. 2022). Measuring sampling biases is thus essential to selecting effective survey methods and interpreting ecological community data.

A major barrier to understanding taxonomic sampling biases is that measuring bias in an absolute sense is often impossible for mobile organisms outside of artificially assembled communities of known composition (e.g., Biro 2013). Instead, practitioners must measure biases of methods relative to one another by evaluating how properties of the samples they produce differ. However, even comparison of methods' relative biases is made complicated by the fact that methods can differ not only in the relative abundances of the organisms they sample (i.e., their bias), but also in the overall number of individuals sampled (sample abundance) (Ramírez‐Hernández et al. 2018; Prendergast et al. 2020; Kuhlman et al. 2021). Raw abundances of individuals and dissimilarity metrics that are sensitive to abundance are commonly used to compare samples from different sampling methods and evaluate relative biases (e.g., Doxon et al. 2011; O'Connor et al. 2019; Checa et al. 2019). However, these analyses risk conflating sampling bias—differences in *relative* abundances of taxa in samples collected by different methods—with sample abundance.

Wild bees are a highly speciose group of insects with diverse ecological traits, posing a challenge for community sampling. Bees' role as pollinators of crops and wild plants, together with evidence of pollination deficits and declines in bee abundance, has motivated widespread community sampling efforts and a large body of research on patterns of bee abundance and species composition across environmental gradients (Woodard et al. 2020). Local bee communities often consist of dozens to hundreds of species (e.g., Giles and Ascher 2006; Ascher et al. 2014; Carril et al. 2018; Rowe et al. 2024) that may vary drastically in body size, the types of flowers they visit, the locations where they nest, and other physical traits and behaviors that may affect their detectability by different sampling methods. Wild bee surveying efforts are dominated by three lethal sampling methods—pan traps, blue vane traps (hereafter “vane traps”), and hand netting—that capture bees by inherently different mechanisms. Comparing the bee communities collected by these methods is essential to designing effective sampling protocols and to understanding the results and limitations of community analyses, particularly for composite datasets that combine data from studies and time periods with different sampling practices (e.g., in comparisons of modern and historic collections; Colla et al. 2012; Bartomeus et al. 2013).

All three common bee‐sampling methods have been the subject of a rich comparative literature. Sampling of bees by hand‐held nets (hereafter “hand netting”) dominated historic collections and continues to be a common component of modern community sampling. This method requires observers to actively seek out bees and capture them with an insect net, typically as the bees are foraging on flowers. Such sampling is subject to the biases of individual observers, which may vary with skill and experience: experts, for instance, tend to detect cryptic and rare taxa more readily than novice observers (Westphal et al. 2008). Larger and more visually conspicuous taxa, such as bumble bees (*Bombus* spp.) are often well‐sampled by hand netting—often more so than by traps—whereas small‐bodied taxa may be relatively underrepresented in nets (Grundel et al. 2011; Rhoades et al. 2017; Pei et al. 2022). In comparison with pan traps, nets have also been found to excel in collecting other strong‐flying taxa such as cellophane bees (*Colletes* spp.) that are more challenging to capture in traps (Wilson et al. 2008; Grundel et al. 2011). Lastly, hand netting is sensitive to the availability and attractiveness of floral resources since observers are typically restricted to netting bees foraging on accessible flowers (Rhoades et al. 2017; Kuhlman et al. 2021). Since bees vary in their floral preferences, the taxonomic composition of hand net samples will depend on the types of flowers from which specimens are collected and how sampling effort is partitioned among the available floral resources.

In addition to hand netting, modern studies have often depended heavily on pan traps and blue vane traps for lethal sampling of bee communities (Portman et al. 2020; Montero‐Castaño et al. 2022). These methods can capture vast numbers of specimens and often produce greater numbers of species than hand netting (Rhoades et al. 2017; Campbell et al. 2023; Larson et al. 2024), though some studies have found the reverse to be true (e.g., Roulston et al. 2007). In contrast to hand netting, traps capture bees passively, attracting them by mimicking the UV and color patterns of flowers. Bees drawn to the trap are captured in a bowl (for pan traps) or jug (for vane traps), where they drown in soapy water or propylene glycol. In comparison to hand‐netting, passive pan and vane traps are robust to user experience, and trapping protocols are relatively easy to standardize, making these methods an attractive choice for efforts to sample and monitor bee communities (e.g., Droege et al. 2016). Yet, despite the apparent ease of standardizing passive sampling, traps are also sensitive to environmental context: traps compete with flowers to attract foraging bees, so abundant, attractive floral resources nearby may lower trap effectiveness (Chamorro et al. 2023; Pei et al. 2022). Such effects appear to be context‐dependent, however, with other studies finding either positive or non‐significant relationships between floral abundance and the number and diversity of bees caught by traps (Rhoades et al. 2017; Krahner et al. 2024). Traps may also be biased toward collecting bee taxa whose preferred floral resources are similar in color or UV reflectance (Leong and Thorp 1999). Patterns of sampling effectiveness may further differ between trap designs: pan traps are notorious for capturing high numbers of small sweat bees (Halictidae), particularly of the genus *Lasioglossum* (Portman et al. 2020; Pei et al. 2022; Campbell et al. 2023), whereas large bumble bees and longhorn bees that may escape shallow pans are common in vane trap samples (Cane et al. 2000; Joshi et al. 2015; Rhoades et al. 2017; Portman et al. 2020).

Although existing studies have demonstrated that pan traps, blue vane traps, and hand netting often differ in the abundance, richness, and composition of bee taxa they collect, efforts to quantify taxonomic sampling biases have still been limited in several key ways. Firstly, studies have often focused heavily on comparisons of raw abundance and species richness to draw conclusions about which methods are most effective for sampling bee communities (e.g., Cane et al. 2000; Roulston et al. 2007; Hall 2018; Hutchinson et al. 2022). As has previously been acknowledged (e.g., Pei et al. 2022), these metrics are useful for describing community samples but are highly dependent on sampling effort. As a result, raw abundance and richness are difficult to compare between methods that collect bees by inherently different mechanisms where units of effort may be impossible to equalize (e.g., passive traps compared to active hand netting). Commonly used tools for comparing community properties under equalized effort, such as sample‐based rarefaction curves (Westphal et al. 2008; Joshi et al. 2015) and coverage (Chamorro et al. 2023; Campbell et al. 2023), are likewise misapplied in this context where effort is incomparable and where methods may differ in the overall community of bees they are able to sample. Instead, more tractable comparisons can be made by equalizing sample abundance (Larson et al. 2024). Finally, comparing only *absolute* abundances of individual taxa is insufficient to demonstrate differences in sampling *bias*, which pertains to differences in *relative* abundance: a method that samples bees more intensively than another could collect more individuals of a given taxon even if individuals of that taxon make up a relatively smaller proportion of that sample.

Another challenge for many existing analyses of wild bee sampling biases is the use of sampling designs that preclude accurate comparison. Specifically, several previous studies have analyzed unpaired sampling schemes, drawing comparisons between samples collected by methods that were not necessarily used simultaneously (Westphal et al. 2008; Grundel et al. 2011; Nielsen et al. 2011; Prendergast et al. 2020). Bee communities can show substantial variation in abundance and composition over time because of species' phenology, population dynamics, weather, and floral resource availability (Vicens and Bosch 2000; Harrison et al. 2018a). As a result, pairing sampling effort such that compared methods are used to collect bees at the same times and locations is essential to avoid confounding differences in sampling bias with actual changes in the sampled community.

Many recent analyses of wild bee sampling bias have measured this phenomenon by comparing differences in community composition between samples taken by different methods, often using dissimilarity metrics, ordination, and multivariate statistics (Popic et al. 2013; Joshi et al. 2015; Prendergast et al. 2020; Pei et al. 2022; Campbell et al. 2023). These studies have addressed sampling biases more accurately than raw abundance‐focused analyses, showing that samples from common methods such as pan traps and hand netting differ in the relative abundances of bee species, genera, families, and functional groups. However, even comparison of compositional dissimilarity can be limited by differences in sampling intensity between methods. Indeed, frequently used dissimilarity metrics such as Sørenson and Bray–Curtis indices are sensitive to differences in sample size and completeness (Beck et al. 2013), which commonly occur between samples taken by different methods (Roulston et al. 2007; Rhoades et al. 2017; Prendergast et al. 2020). Without making a concrete distinction between differences in composition and sample abundance, it is difficult to conclude whether two methods are likely to yield complementary pools of species. Null modeling approaches to comparing community composition offer a possible solution to this concern (Chase et al. 2011), but to our knowledge have not been applied in the context of measuring sampling biases. Compositional analyses of sampling bias have also rarely reported estimates of effect sizes with confidence intervals for individual groups of bees (but see Tronstad et al. 2022; Krahner et al. 2024). Estimating both effect sizes and confidence intervals for methods' relative sampling biases with respect to specific taxa is essential for both evaluating which taxa are most sensitive to choice of sampling method and measuring the precision of these estimates (Nakagawa and Cuthill 2007).

In this study, we build on the community sampling literature by comparing biases of pan traps, hand netting, and vane traps with a large dataset of bee community studies, all of which used paired sampling designs such that bees were captured using the various methods at the same places and times. In comparing richness and composition between methods, we carefully control for raw sample abundance to gain clear inference about how these community characteristics differ. Additionally, we use a novel linear modeling approach to quantify the relative biases of methods in our dataset with respect to individual genera and size classes of bees, adding to previously described patterns with greater resolution about which taxa are most sensitive to sampling choices. We ask whether (1) species richness and (2) species composition of bee community samples differs between sampling methods and (3) whether bees of particular genera or body sizes are collected disproportionately by different sampling methods. In doing so, we develop and demonstrate a comprehensive system to compare taxonomic sampling biases for wild bees and similarly diverse communities of organisms that recognizes bias as a distinct issue from sampling effectiveness (i.e., from raw sample abundance).

## 2 Methods

### 2.1 Data Collection and Processing

We used data from 5 years of previous studies conducted in the mid‐Atlantic region of the United States (Winfree et al. 2014; Harrison et al. 2018a, 2018b; Smith et al. 2021; T. Harrison, unpublished data; Table S1). The paired sampling designs used by these studies afforded two types of methodological sampling comparisons: comparison of pan traps against blue vane traps (3 years of data) and comparison of pan traps against hand netting (2 years of data). Pan traps were deployed as arrays or linear transects of alternating white‐, yellow‐, and blue‐painted plastic bowls filled with water and a drop of dish soap to break surface tension. In paired pan trap and vane trap sampling, one or more blue vane traps filled with soapy water were deployed immediately adjacent to each pan trap array; pan and vane traps were set up and taken down at approximately the same time on each sampling occasion. Numbers and exact placement of pan and vane traps varied between the three studies, though in all cases many more pan traps were used per sampling event than vane traps (39 pan traps and four vane traps by Harrison et al. 2018a, 2018b; 24 pan traps and two vane traps by Smith et al. 2021). However, the relative numbers of pan traps and vane traps and placement of traps were constant across sampling events within each study (Data S1). In paired pan trap and hand net sampling, collectors sampling with hand nets walked freely about in the vicinity of the pan trap array and netted bees from flowering plants. Importantly, both methods in any given sampling event were used within the same 24‐h period to standardize weather, bee and flower phenology, and other variables that may affect wild bee activity and detection of individual taxa.

**FIGURE 1:**
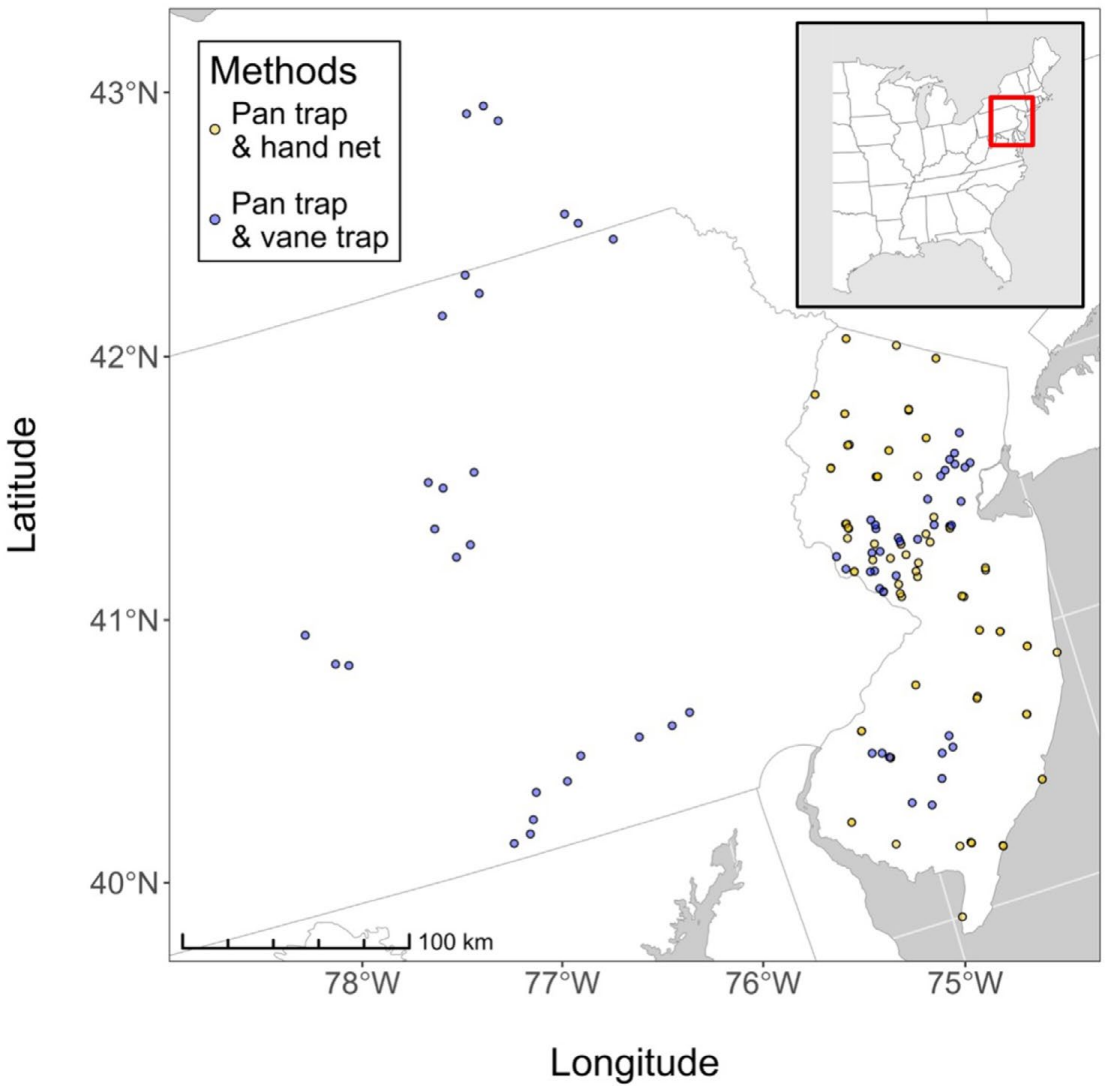
Study sites. We used data from a set of five studies across New Jersey, Pennsylvania, and New York. Yellow points show sites where pan trap sampling was paired with simultaneous sampling by hand net, whereas blue points show sites where pan trap sampling was paired with vane traps.

Bee specimens were identified in the lab using published revisions and taxonomic keys (Data S2), primarily by professional taxonomists. Over 99% of specimens were identified either to species or to one of four species complexes that cannot be resolved at present based on morphology alone (Table S2). Individuals belonging to these four species complexes accounted for about 7.4% of all specimens. The small proportion (~0.8%) of specimens that could not be determined to a species or species complex were excluded from the final dataset we analyzed here. Honey bees ( *Apis mellifera* ) were also excluded from the final dataset because they are primarily a managed species in our study region and are therefore distinct from the communities of wild bees we aimed to analyze. One study in the dataset (T. Harrison, unpublished data) intentionally avoided collecting bumble bees (*Bombus* spp.) by hand net, so we removed all remaining incidental bumble bee specimens from this study prior to analysis. The final composite dataset included 27,485 specimens collected at 118 sites in 443 unique sampling events between April 2006 and May 2018 (Figure 1, Table S1).

### 2.2 Does Richness of Bee Community Samples Differ Between Methods?

For some community‐sampling objectives, such as inventory, efficient sampling methods detect high numbers of species while sampling as few individuals as possible. To test which methods accumulate species most efficiently, we compared richness between them by plotting an individual‐based rarefaction curve for each method, pooled across all sampling events. Individual‐based rarefaction curves illustrate the rate of increase in species richness as the number of individuals sampled increases; richer samples thus produce taller rarefaction curves. Sample‐based rarefaction, which estimates how species richness increases with the number of samples collected, is inappropriate for our purpose of comparing richness between sampling methods because the amount of effort contributed by a single sample is not consistent across methods that sample bee communities with different mechanisms and intensities.

We constructed rarefaction curves for each method up to the observed sample richness using estimates of interpolated richness and 95% confidence intervals calculated with the function ‘estimateD’ from the R package *iNEXT* version 3.0.1 (Chao et al. 2014; Hsieh et al. 2016). We determined whether sample richness differed between sampling methods by comparing their respective rarefaction curves at the sample size of the method that caught fewer individual bees. We concluded that sample richness differed significantly between methods if their 95% confidence intervals at that point on the curve did not overlap. Although it is often preferable to compare community richness at equalized sample *completeness* (i.e., coverage; Roswell et al. 2021), we find it more relevant and interpretable to equalize sample *size* in this analysis because the definition of the “complete” community of bees at a site could differ between methods that effectively sample different communities (e.g., because the methods attract different bee species). To explore the possibility that rarefaction curves derived from pooled datasets for each type of sampling comparison could be skewed by individual studies that used especially unequal levels of sampling effort between methods and/or included inordinately species‐rich sites, we also plotted curves for pairs of sampling methods within each individual study.

### 2.3 Does Composition of Bee Community Samples Differ Between Methods?

#### 2.3.1 Measuring Community Dissimilarity

Even when two methods are used simultaneously to sample a given bee community (i.e., paired sampling), those methods may effectively sample *different* pools of bee species if they differ substantially in their ability to detect those bees (Prendergast et al. 2020; Kuhlman et al. 2021; Pei et al. 2022). However, differences in species composition between samples are often difficult to measure independently of differences in sample abundance and richness (Beck et al. 2013). In order to overcome this issue, we measure compositional dissimilarity between samples collected by different methods using the Raup–Crick metric. Raup–Crick dissimilarity calculates differences in community composition while accounting for differences in communities' individual richness by comparing the observed number of species shared between two samples against a null distribution of the expected number of species shared between two samples with the same richnesses (Chase et al. 2011). For any pair of samples, this null is produced by calculating the number of species shared between two random draws of species with the same richnesses as the “real” samples. These simulated draws are taken from the full pool of species observed across all samples, with each species' probability of being drawn equal to the proportion of samples it occurred in. Dissimilarity is then calculated as the proportion of random draws with as many shared species as the observed pair of samples or fewer. This metric is sensitive mostly to differences in the frequency of common species in the compared samples and is less sensitive than other metrics (such as Sørenson and Bray–Curtis) to differences in sample size—a property that we find ideal for comparing methods that sample bee communities with immeasurably different intensities. For each type of sampling comparison, we calculated Raup–Crick dissimilarity between all pairs of samples using the function ‘raup_crick’ provided by Chase et al. (2011).

#### 2.3.2 Comparing Community Composition

To statistically test whether species composition differed systematically between pairs of methods in each sampling comparison, we use permutational analysis of variance (PERMANOVA) via the function ‘adonis2’ from R package *vegan* version 2.6.10 (Oksanen et al. 2025) with sampling method as the categorical grouping variable. Specifically, this test uses a distance matrix to calculate the ratio of compositional variation among samples collected by the same method to variation among samples collected by different methods. Statistical significance is determined by comparing this value against a null distribution produced by repeatedly assigning samples randomly to each method and recalculating this ratio. We included “study” as an additional predictor in the PERMANOVA model for both sampling comparisons to account for differences in exact methodology between studies (e.g., numbers of traps used). Since we compare sample composition with Raup–Crick dissimilarity, this analysis effectively answers the question “Are samples collected using the same method more likely to have been drawn from the same species pool than samples collected by different methods?”. Dissimilarities between samples were visualized with nonmetric multidimensional scaling (NMDS) using the function ‘metaMDS’ from *vegan*.

### 2.4 Are Bees of Particular Genera or Body Sizes Collected Disproportionately by Different Sampling Methods?

**FIGURE 2:**
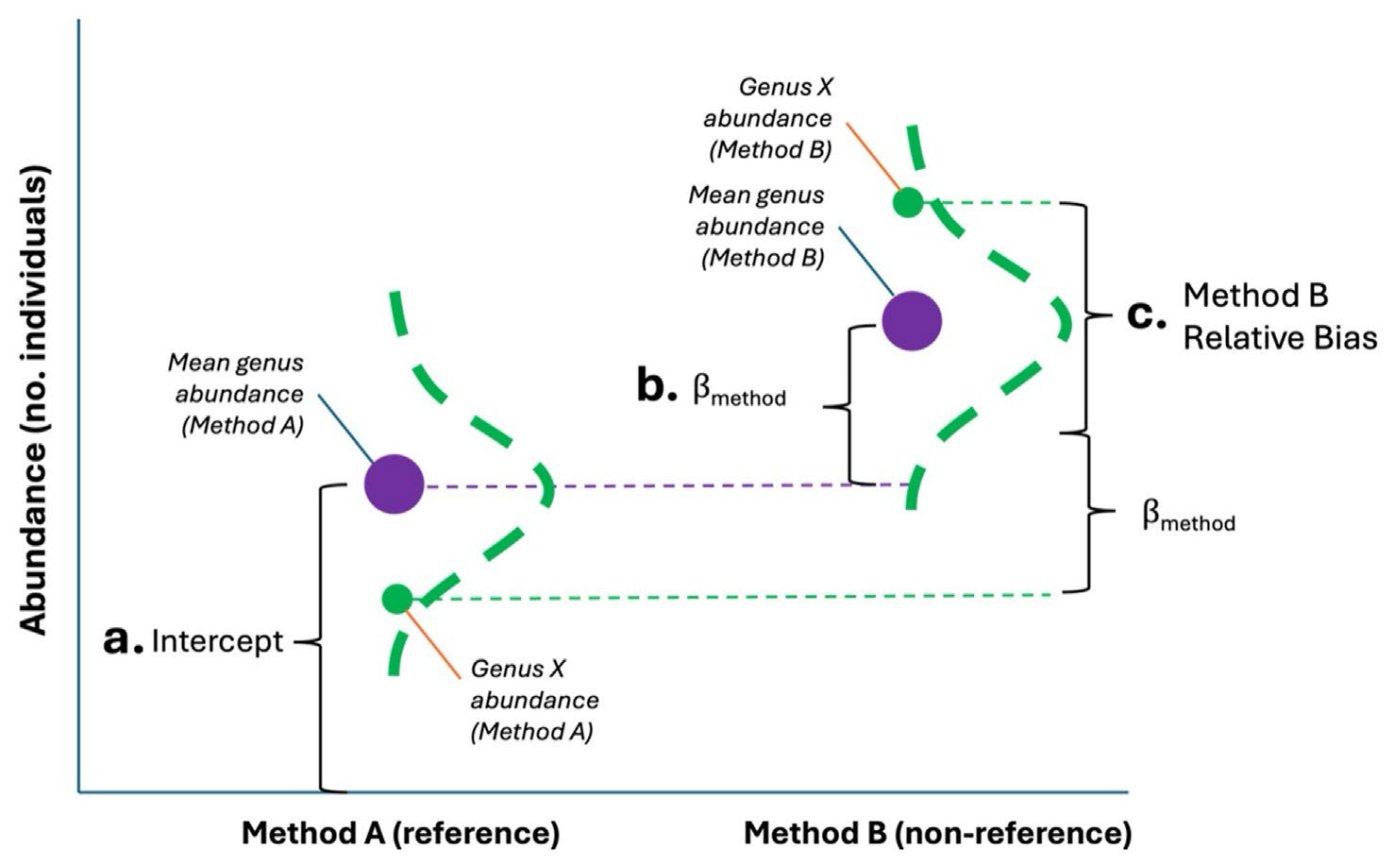
Quantifying relative sampling bias with generalized linear mixed models. In this hypothetical implementation of our sampling bias model, the (a) model intercept describes the mean number of individuals collected per genus in a single sampling event (site‐date) using Method A—the “reference” that an alternative method will be compared against. The (b) fixed effect of sampling method estimates the mean difference between Method A and Method B in the number of specimens collected per genus in a single sampling event. Finally, the (c) random intercept and slope distribute abundances of individual genera around the mean genus abundance in each method and modify the effect of sampling method for each genus. Here, the mean number of individual bees per genus is greater in Method B than in Method A (β_method_ is positive). Furthermore, the mean abundance of bees of Genus X per sample increases even more than that of the average genus when sampling is performed with Method B rather than Method A, suggesting that Method B is relatively biased *toward* sampling Genus X relative to Method A.

We quantified taxonomic and trait biases for each sampling method using negative binomial generalized linear mixed models implemented with the function ‘glmmTMB’ (package *glmmTMB*, version 1.1.10; Brooks et al. 2017) (Figure 2). Specifically, we asked whether different bee genera and genera belonging to different body size classes showed significantly above‐ or below‐average change in abundance between samples collected by different methods. We tested taxonomic biases by aggregating bee abundances at the genus level because sample sizes for most individual species were too small for robust analysis. To ensure robust comparison of bee genus relative abundances between methods, we restricted each sampling comparison to include only common “focal genera” that were captured by at least one method on at least 10 of the sampling events when that pair of methods was used together. This constraint allowed us to analyze abundances of 21 genera in comparison of pan traps and hand netting, and 20 genera in comparison of pan traps and vane traps. Each type of sampling comparison analyzed most of the same focal genera, with only five genera that were unique to one comparison or the other (*Augochloropsis*, *Heriades*, and *Xylocopa* were included only in the comparison of pan traps and hand netting, whereas *Anthophora* and *Eucera* were analyzed only in the comparison of pan traps and vane traps). Even when using this arbitrary constraint to exclude especially rare and poorly sampled genera, we retained about 99% of specimens from each of the two sampling comparison datasets for analysis.

For each sampling comparison (pan vs. net and pan vs. vane), we calculated the number of individuals from each focal genus collected by each method in each sampling event (site‐date). We then modeled abundances of genera as a function of sampling method for each of the two sampling comparisons, with genus included as a random intercept to account for differences in abundance among genera and method as a random slope to allow differences in responses of genera to sampling method. We included an additional random intercept of sampling event (site‐date) to account for spatial and temporal variation in overall bee abundance. Overall differences in the number of bees captured by each method (i.e., sample abundance) are modeled here by the fixed effect of sampling method, which estimates how much the abundance of individual genera differs on average between sampling methods (on the logarithmic scale).

We measure relative sampling biases of each method for individual genera using the random slope with respect to the method. This term quantifies methods' relative biases for each genus by estimating how much the log‐scale difference in a genus' abundance between sampling methods deviates from the average change in log abundance per genus. In other words, this term is *not* an estimate of change in abundance between methods, but rather a genus‐specific adjustment to the average change (Figure 2). In the glmmTMB syntax, this model is written as “abundance_genus_ ~ method + (method|genus) + (1|sampling event)”. To standardize the interpretation of analyses, we defined pan traps as the reference level in all models so that coefficients are expressed as relative biases of hand net and vane trap samples compared to pan traps. Therefore, when interpreted on the log scale of the negative binomial model, relative bias quantified by the random slope term is positive for genera that are overrepresented in hand net or vane trap samples relative to pan trap samples, and negative for genera that are underrepresented in hand net or vane trap samples relative to pan traps. We concluded that a pair of sampling methods differed significantly in their biases with respect to a given genus if conditional 95% confidence intervals around the random slope estimate for that genus did not overlap with zero. Mixed model fits were confirmed by analyzing model residuals with the function ‘simulateResiduals’ from R package *DHARMa* (version 0.4.7; Hartig 2024).

To test whether bee genera that were close in size were affected similarly by choice of sampling method, we first categorized genera according to female intertegular distance (ITD; a standard metric of bee body size; Cane 1987). Using an existing set of specimen ITD measurements (Bartomeus et al. 2013) filtered to include only species that occurred in our composite dataset, we first calculated the mean female ITD across sampled specimens for each bee species. We then calculated mean female ITD for each genus by averaging the mean female ITD of each species in the genus. After removing the genus *Xylocopa* (an outlier with an ITD over 50% larger than that of the next largest genus), we categorized genera by dividing the range of average female ITD measurements across genera into four bins of equal breadth. We assigned *Xylocopa* to the largest size bin *post hoc*. From smallest to largest, we refer to these size classes as “small” (1.04–1.76 mm), “medium” (1.77–2.48 mm), “large” (2.49–3.20 mm), and “very large” (3.21–5.91 mm) (Table S3).

We then fit a pair of models (one for each sampling comparison) analogous to those used to quantify genus‐level relative biases, but using body size class (rather than genus) as the random intercept term. Similarly to the genus‐level model, a random slope with respect to sampling method quantifies methods' relative bias for each size class of genera. This coefficient is positive for size classes that are relatively overrepresented in net and vane samples and negative for size classes that are relatively underrepresented by those methods. We concluded that the relative biases of sampling methods with respect to genera of a given body size class differed significantly if conditional 95% confidence intervals around the random slope estimate for that group did not overlap with zero. In glmmTMB syntax, this model is written as “abundance_genus_ ~ method + (method|body size class) + (1|sampling event)”.

## 3 Results

### 3.1 Does Richness of Bee Community Samples Differ Between Methods?

**FIGURE 3:**
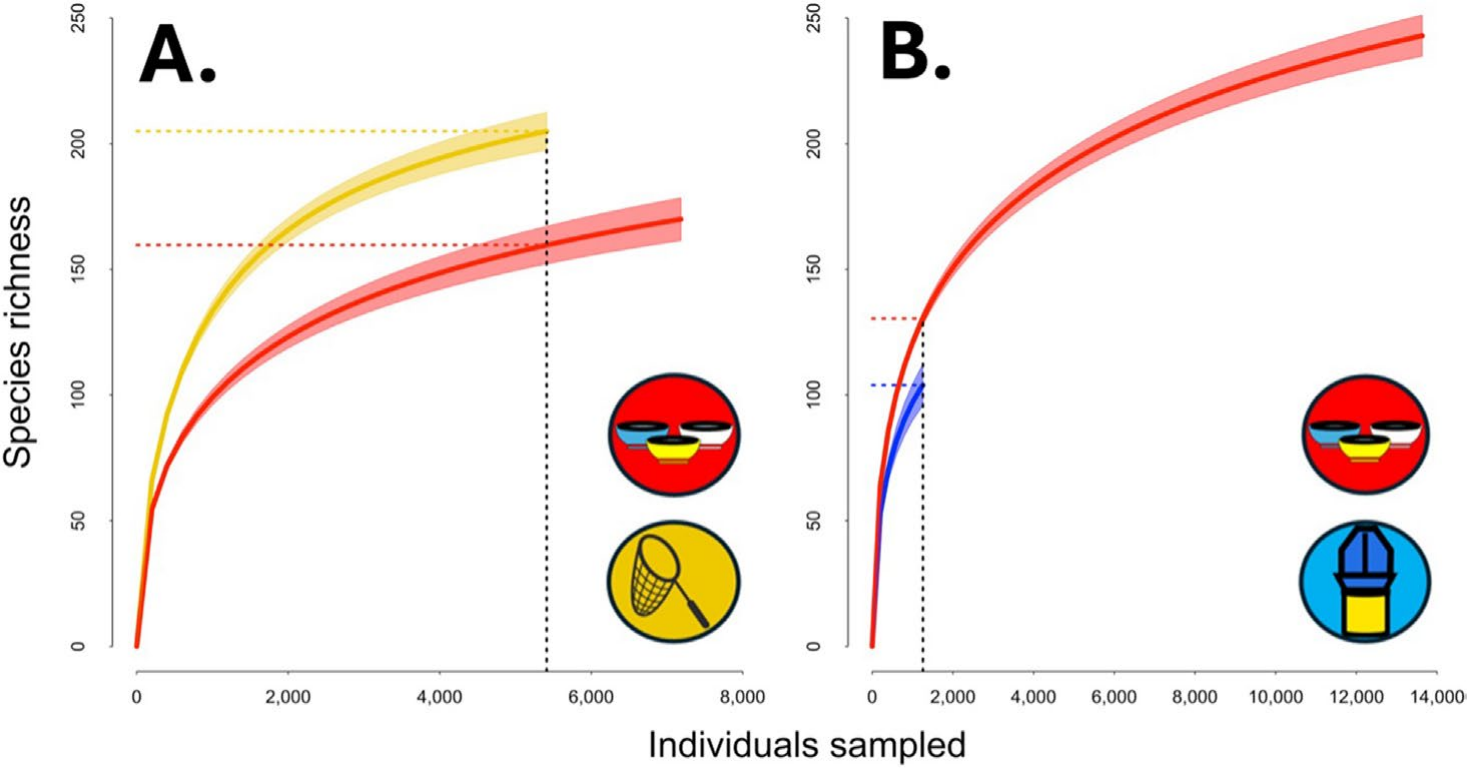
Rarefaction curves and comparison of species richness for pan trap (red) and (A) hand net (yellow) and (B) vane trap (blue). Curves show rarefied estimates of sample richness, with shaded 95% confidence intervals. Vertical dashed lines (black) indicate the number of individual bees captured by the method with the lower sample size in each pair, whereas colored horizontal dashed lines indicate the richness of samples from each method at that sample size. Lack of overlap in 95% confidence intervals at this point is interpreted as a significant difference in richness between methods.

Paired hand net and pan trap sampling captured a total of 12,596 individual bees across all sampling events (hand net: 5412 individuals; pan trap: 7184 individuals). In total, 205 species from 34 genera were represented in hand net sampling, whereas pan traps detected 170 species from 30 genera. Twenty‐seven genera were shared between samples collected by these two methods. When sample sizes were equalized between these methods, predicted hand net sample richness (205, CI_95_ 197–213) was significantly greater than for pan traps (160, CI_95_ 153–167) (Figure 3A). Results were qualitatively similar when rarefaction curves were made for each of the three studies independently (Figure S1).

Paired vane trap and pan trap samples captured a total of 14,889 individual bees (pan trap: 13,632 individuals; vane trap: 1257 individuals). Although the number of pan traps used at each site greatly exceeded the number of vane traps (Data S1), the average number of bees captured per trap per sampling event was very similar between the two methods (vane: 1.9 bees per trap, range 0–25 bees per trap; pan: 1.7 bees per trap, range 0–20 bees per trap). Pan traps captured 243 species representing 35 genera, whereas 104 species of 23 genera were captured by vane traps. All 23 genera captured by vane traps were also represented in pan trap samples. After equalizing pan trap abundance to match vane traps, predicted sample richness was significantly greater for pan traps (131, CI_95_ 128–133) than for vane traps (104, CI_95_ 95–113) (Figure 3B). A qualitatively similar pattern was found when rarefaction curves were calculated for each of the three individual studies in this comparison, though predicted sample richness between pan and vane traps differed significantly only for the single study in which the vane trap sample size exceeded 500 individuals (compared to thousands of individual bees sampled by pan trap in each individual study) (Figure S2 and Table S1).

### 3.2 Does Composition of Bee Community Samples Differ Between Methods?

**FIGURE 4:**
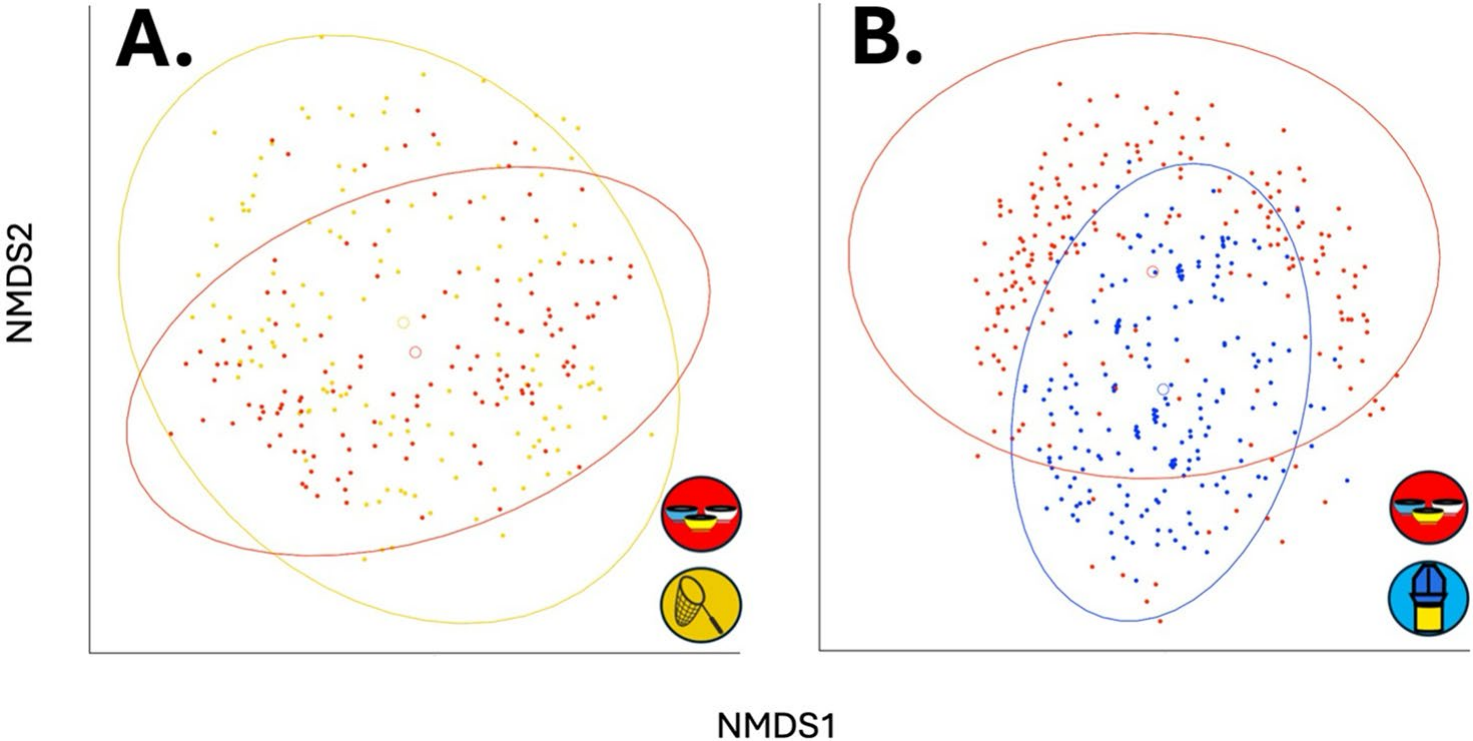
Nonmetric multidimensional scaling of Raup–Crick dissimilarities between samples collected by paired bee community sampling methods. Individual points represent samples collected by a single method on a single sampling event (site‐date combination) by pan traps (red) and (A) hand net (stress = 0.27) and (B) vane trap (stress = 0.28). Group centroids are represented as open circles, surrounded by 95% confidence ellipses of the same color. Hand netting sampled a distinct pool of bee species compared to pan traps (*p* < 0.001, F = 61.55), as did vane traps (*p* < 0.001, F = 39.98).

The pool of species collected differed significantly between hand net and pan trap samples (PERMANOVA; *p* < 0.001, F = 61.55) (Figure 4A), as well as between samples collected by pan trap and vane trap (*p* < 0.001, F = 39.98) (Figure 4B).

### 3.3 Are Bees of Particular Genera or Body Sizes Collected Disproportionately by Different Sampling Methods?

**FIGURE 5:**
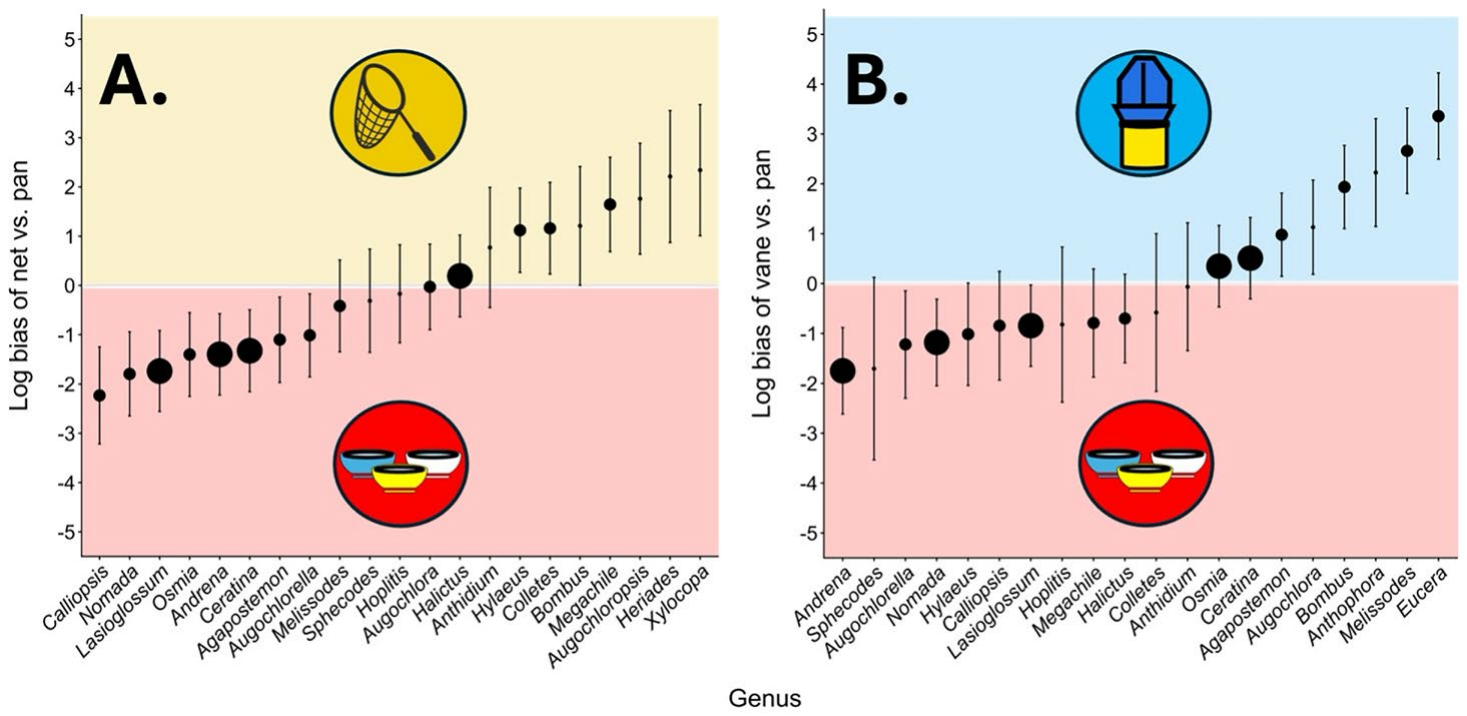
Estimated relative genus‐level biases of common sampling methods. Points show biases of hand net and vane trap sampling relative to pan trap sampling for each bee genus as log‐scale random slope estimates from a negative binomial generalized linear mixed model with 95% confidence intervals. This coefficient estimates how much the difference in sample abundance between methods for bees of each genus deviates from the average difference in abundance across all genera. Genera that were overrepresented in (A) hand net or (B) vane trap samples relative to pan traps have a positive bias on the log scale, whereas those that were relatively underrepresented by hand netting or vane traps have a negative log bias. Point sizes correspond to the order of magnitude of abundance of each genus in each comparison (tens, hundreds, or thousands).

Abundances in paired pan trap and hand net samples were compared for 21 genera (Figure 5A and Table S4). Pan trap samples relatively over‐represented eight genera (*Calliopsis*, *Nomada*, *Lasioglossum*, *Osmia*, *Andrena*, *Ceratina*, *Agapostemon*, and *Augochlorella*). Another seven genera (*Xylocopa*, *Heriades*, *Augochloropsis*, *Megachile*, *Colletes*, *Bombus*, and *Hylaeus*) were relatively over‐represented by hand net as compared to pan trap. Abundances of 20 genera were compared between paired pan trap and vane trap samples (Figure 5B and Table S4). Pan traps relatively over‐represented the genera *Andrena*, *Augochlorella*, *Nomada*, and *Lasioglossum*, whereas vane traps relatively over‐represented *Eucera*, *Melissodes*, *Anthophora*, *Bombus*, *Augochlora*, and *Agapostemon*.

**FIGURE 6:**
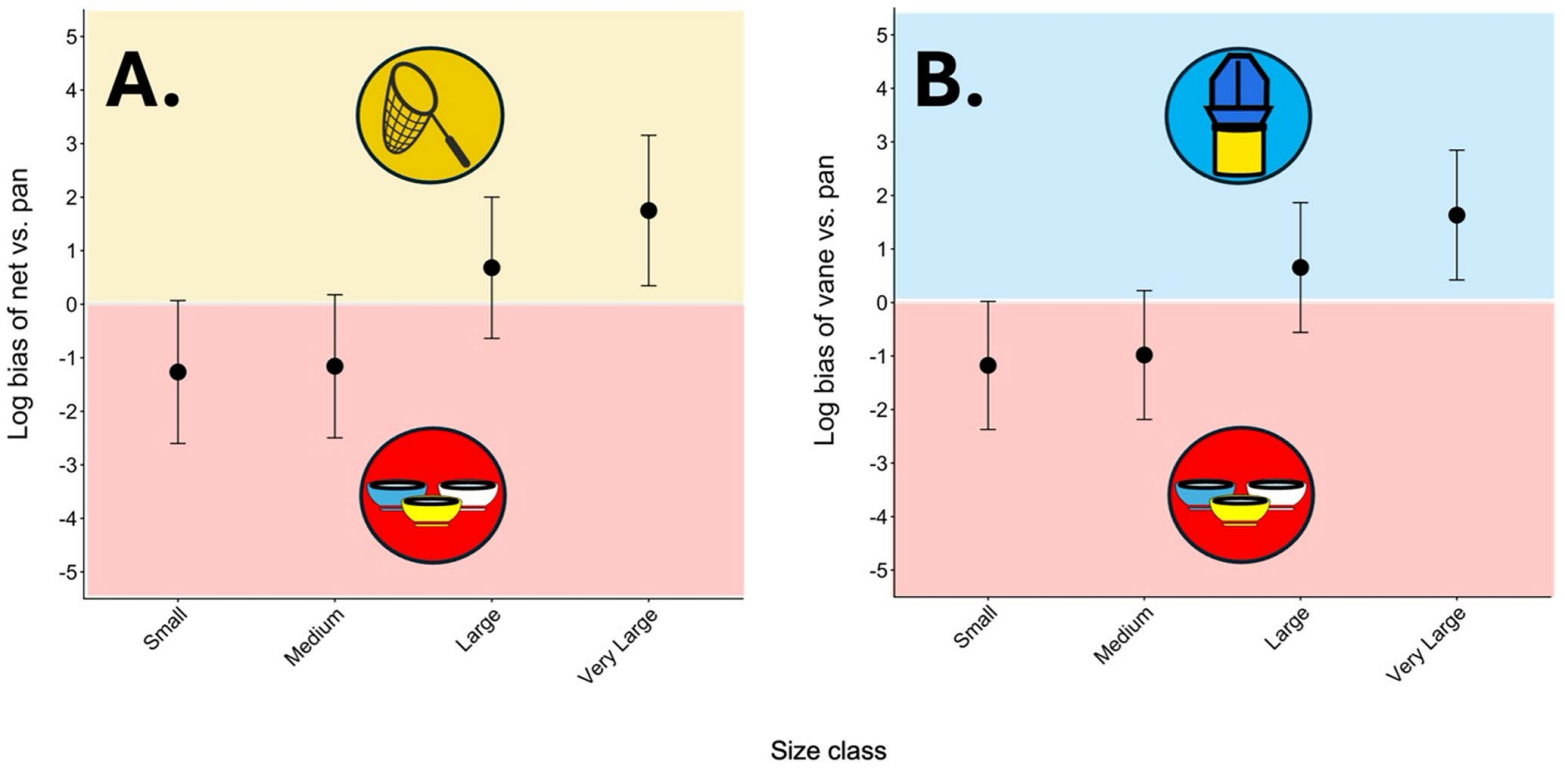
Estimated relative body size biases of common sampling methods. Points show biases of hand net and vane trap sampling relative to pan trap sampling for each size class of bees as log‐scale random slope estimates from a negative binomial generalized linear mixed model with 95% confidence intervals. This coefficient estimates how much the difference in sample abundance between methods for bees of each size class deviates from the average difference in abundance across all size classes. Size classes of bees that were overrepresented in (A) hand net or (B) vane trap samples relative to pan traps have a positive bias on the log scale, whereas those that were relatively underrepresented by hand netting or vane traps have a negative log bias.

Relative to both hand netting and vane traps, pan traps tended to underrepresent the largest size class of bee genera (“very large”). Other size classes of genera (“small,” “medium,” and “large”) showed no significant systematic difference in bias between either pair of sampling methods (Figure 6 and Table S5).

## 4 Discussion

### 4.1 A Framework for Measuring Taxonomic Sampling Biases

Measuring the taxonomic biases of community sampling methods is an important step in interpreting the samples they produce, but quantifying bias and sample abundance as separate properties has remained a challenge for comparative studies (Pei et al. 2022). For wild bees, an especially speciose and ecologically and economically significant group of insects, extensive efforts have shown that common sampling methods can differ in the richness and overall composition of taxa they collect (Popic et al. 2013; Joshi et al. 2015; Prendergast et al. 2020; Pei et al. 2022; Campbell et al. 2023). Despite this, few methodological studies have tested differences in community characteristics in ways that are not sensitive to differences in sample abundance or estimated the magnitude of the effect of sampling method on relative abundances of specific bee taxa. Here, we build on the existing literature by comparing three common sampling methods in ways that explicitly consider differences in sample abundance between methods. We also provide some of the first estimated effect sizes for methods' relative biases with respect to individual genera and size classes of bees. The comparisons we make are facilitated by the paired sampling designs used to gather our datasets—a necessity for accurate methodological analyses. Altogether, our study increases the resolution of understanding of sampling biases for wild bees and, for datasets of simultaneous sampling effort by multiple methods, the series of analyses we apply here illustrates a replicable framework for careful comparison of community sample characteristics that accounts for abundance.

Our modeling approach was able to quantify the relative biases of methods for individual bee genera and size classes, even when those biases ran counter to immense differences in sample abundance. Pan traps, for instance, captured over 10 times as many bees as vane traps, with all but three genera captured in greater abundance by pans. Nevertheless, we were able to discern that even some genera like *Augochlora* and *Agapostemon* that were over three times as *abundant* in pan traps as compared to vane traps were, in fact, relatively *under*represented by pans. By measuring both the magnitude and precision of estimates of methods' relative biases for individual genera, we provide an important advance for methodological studies. From these taxon‐specific estimates of relative biases, we can discern that the greatest differences in bias between methods affected some of the most abundant and species‐rich bee genera in our dataset, such as *Lasioglossum*, *Andrena*, and *Nomada* (Figure 5). This is a significant detail for interpreting community sampling studies since abundance and diversity of common, speciose bee taxa can be important drivers of community‐wide patterns in abundance, richness, diversity, and even ecosystem services (Kleijn et al. 2015). Targeted monitoring efforts (e.g., for estimating occupancy of particular species; Otto et al. 2025) also benefit from a taxon‐specific understanding of sampling bias because identifying methods that best sample the target taxon could facilitate efficient sampling while minimizing the relative abundance of bycatch. However, we caution against extrapolating the exact effect sizes of biases we report for bee genera to sites outside of our study area. Several prior reports have found indications that the exact performance of sampling methods can vary (Leong and Thorp 1999; Prendergast et al. 2020; Chamorro et al. 2023; Pei et al. 2022), differing among habitat types, floral contexts, and among bee species within genera. Rather than assuming the specific numerical differences in bias we find, we urge practitioners to repeat our analytical methods to discern and interpret biases in their own paired sampling datasets.

We found widespread variation in taxonomic biases among bee sampling methods, with 80% of genera showing significant differences in relative abundance in at least one sampling comparison. The specific patterns of bias we detect vary in their consistency with previous methodological analyses. Some patterns support conclusions that other studies have also reached almost unanimously, such as the association of *Lasioglossum* sweat bees with pan traps (Westphal et al. 2008; Grundel et al. 2011; Joshi et al. 2015; Hutchinson et al. 2022; Campbell et al. 2023). We also corroborate a positive bias of vane traps for collecting *Agapostemon* and the longhorn bee genera *Melissodes* and *Eucera* (Joshi et al. 2015; Rhoades et al. 2017). For other bee taxa, known patterns of sampling bias are less clear. Bees in the families Apidae and Megachilidae, for instance, have been found to be most strongly represented in pan trap samples in some studies (Grundel et al. 2011) and in net samples in others (Pei et al. 2022). These family‐level analyses of sample composition may be excessively coarse, as we found that genera belonging to the same family were sometimes subject to opposite biases from a given pair of methods. Among megachilid bees, we find that pan traps are biased toward collecting *Osmia* relative to hand netting but are relatively biased against collecting *Megachile*. The same was true of apid bees, where *Xylocopa* and *Bombus* were better represented in hand netting relative to pan traps, even though closely related *Ceratina* were better represented in pans. Overall, we found that pan traps tended to have poorer representation of the largest bee genera relative to hand netting and vane traps, supporting the idea that varying patterns of bias within bee families may be driven in part by differences in body size (Rhoades et al. 2017; Pei et al. 2022).

Differences in taxonomic bias among the methods we analyzed were also evident in the species composition of the samples they collected, with pan traps capturing a distinct pool of bee species as compared with either hand netting or vane traps. Our findings in this regard echo several existing studies that have found compositional differences in the samples collected by these same methods (Joshi et al. 2015; Rhoades et al. 2017; Tronstad et al. 2022; Pei et al. 2022). We argue, however, that our comparison of species composition among these methods is more straightforward to interpret because of our use of the Raup–Crick metric, a null model‐based measure of dissimilarity (Chase et al. 2011). Although still an infrequently used tool among ecologists, this metric's null modeling approach quantifies variation in species composition while being relatively insensitive to the richness and abundance of individual community samples. The robustness of the Raup–Crick metric to differences in sample size offers an advantage when comparing methods that sample with different intensities, allowing us to more directly test how methods differ in the species they sample and further tease apart biases irrespective of overall sample abundances.

Pairs of methods in each sampling comparison captured different numbers of species, even when compared at the same sample abundance. For a given number of individuals sampled, hand netting captured a greater number of species relative to pan traps, whereas pan traps captured a greater number of species relative to vane traps. We compare richness among samples because it is a commonly interpreted measure in community ecology, as well as a pertinent metric of efficacy for studies focused specifically on faunal inventory. Methods also differed, sometimes greatly, in the amount of effort required to capture a given number of *individuals*—an hour of netting by one collector, for instance, captured as many bees as 68 pan traps in the same amount of time, whereas vane traps caught 1.5 times as many bees per trap per hour as pan traps. However, although differences in overall sample abundance between methods have sometimes been compared as a measure of relative effectiveness or performance (Cane et al. 2000; Roulston et al. 2007; Hall 2018; Hutchinson et al. 2022), we caution that this may be misleading when the methods compared have incomparable units of sampling effort, as is the case between hand‐netting and passive trapping, or between different trap types.

### 4.2 Implications of Sampling Bias for Monitoring Wild Bees

Our findings suggest that sampling entire bee communities in a consistent and unbiased fashion is a challenging task. As others have acknowledged, a possible way forward for investigating ecological questions that require bee community data might be to employ multiple sampling methods with “complementary” biases simultaneously (Cane et al. 2000; Roulston et al. 2007; Wilson et al. 2008; Rhoades et al. 2017; Pei et al. 2022; Bell et al. 2023). We agree with the use of multiple sampling methods as a solution to sampling biases in inventory efforts whose goal is to simply document the bee taxa present at a given place and time. Since the detectability of bee taxa varies between methods, increasing the number of sampling methods used in a study is likely to produce greater species richness (Bell et al. 2023). It is less clear, however, if simultaneous use of multiple sampling methods should decrease the effects of bias for studies concerned with the relative *abundances* of taxa, as is commonly the case in community ecology. Because the true composition of a sampled ecological community is unknown, one cannot be certain that including an additional method will cause samples to more closely resemble the actual underlying community. Nevertheless, we encourage lethal community sampling efforts to use multiple methods in tandem to verify that analytical results are robust to the choice of sampling method(s). Lethal community‐level data collection may also be improved by adopting standardized sampling protocols, a useful strategy for maximizing the quality and comparability of data across studies even when the methods involved produce biased collections of bee taxa (Levenson et al. 2025).

In addition to using multiple methods and standardizing sampling protocols, practitioners might also consider that some avenues of wild bee research do not require community‐wide data. Although community sampling methods are a popular approach to studying and monitoring wild bee populations, uncertainty remains over how abundances of bees in community samples relate to true population sizes (Briggs et al. 2022). The prevalence of lethal community‐wide sampling in bee monitoring efforts has also raised concerns over the challenges of accurate specimen identification, as well as the taxonomic bottleneck created when large collections of specimens must be identified by a small number of experts (Portman et al. 2020; Tepedino and Portman 2021). Although moderate lethal sampling has not been found to affect entire bee communities (Gezon et al. 2015), collectors must also weigh the possible effects of sampling on rare or threatened bee populations, particularly as these species gain legal protection (Montero‐Castaño et al. 2022; Smith et al. 2025). Practitioners interested in studying population sizes and dynamics, resource requirements, movement, monitoring of specific focal taxa, or other topics that are not inherently based in community ecology might therefore consider gathering data through alternative frameworks, such as population‐level studies (Dorian et al. 2024) or occupancy‐focused monitoring (Otto et al. 2025). By investing in a broad and diverse range of methods for studying wild bees, researchers can complement and confirm conclusions from classic community sampling and help facilitate well‐informed monitoring and conservation.

## Author Contributions

**Max W. McCarthy:** conceptualization (equal), formal analysis (lead), methodology (lead), writing – original draft (lead). **Dylan T. Simpson:** conceptualization (equal), formal analysis (supporting), methodology (supporting), writing – review and editing (equal). **Andrew H. Aldercotte:** conceptualization (equal), formal analysis (supporting), methodology (supporting), writing – review and editing (equal). **Colleen Smith:** data curation (equal), funding acquisition (equal), writing – review and editing (equal). **Tina Harrison:** data curation (equal), funding acquisition (equal), writing – review and editing (equal). **Rachael Winfree:** conceptualization (equal), data curation (equal), funding acquisition (equal), methodology (supporting), writing – review and editing (equal).

## Funding

This work was supported by Rutgers, The State University of New Jersey. National Institute of Food and Agriculture, 1014396 Garden Club of America National Science Foundation Biological Sciences Division of Environmental Biology, 0516205, 0554790.

## Conflicts of Interest

The authors declare no conflicts of interest.

## Supporting information


**Data S1:** Supporting Information.

## Data Availability

Data and code supporting the findings of our study are available on Dryad at https://doi.org/10.5061/dryad.r2280gbr8.
