# Supplementary material for: Separating Sampling Bias From Abundance Shows That Different Methods Catch Different Wild Bees
Source: Ecol Evol. 2026 Feb 12;16(2):e73060. doi: 10.1002/ece3.73060 (PMC12895464; doi:10.1002/ece3.73060)
Supplement: Supplementary file 1 — Data S1: Supporting Information. [file ECE3-16-e73060-s001.docx]

**Methods S1. Sampling methods of individual studies**

The data used in this study was a composite of five bee community datasets collected in New Jersey, New York, and Pennsylvania. Four of these datasets have been analyzed in previous published papers, where they are described in greater detail (Winfree et al. 2014, Harrison et al. 2018a, Harrison et al. 2018b, Smith et al. 2021). The fifth dataset is published and described here for the first time.

Winfree et al. 2014 — pan trap and hand net:

The goal of this study was to determine how patterns of pollinator species loss over a gradient of anthropogenic disturbance related to those species’ linkage in plant-pollinator networks. From this study, we use a dataset of paired pan trap and hand net sampling conducted at 16 sites between April and June of 2006 (13 of the “gradient sites” included in the previously published study, as well as an additional 3 sites in predominately agricultural, suburban, or urban areas that were not used in the original publication). Sampling took place on days with sunny to partly cloudy weather, low winds, and temperatures exceeding 14°C. On each sampling occasion, 3.25-ounce pan traps were deployed for two to four hours in a grid of 39 traps of alternating colors (blue, yellow, and white) spaced 10 meters apart. During this time, hand net sampling of bees on flowers was also conducted for one hour within a 0.5-hectare plot by one to three observers.

Harrison et al. 2018a, Harrison et al. 2018b — pan trap and vane trap:

The first of these studies (Harrison et al. 2018a) aimed to compare abundance, richness, composition, and phenology of bee communities among landscapes dominated by three different habitat types: forest, agriculture, and urban. The second study (Harrison et al. 2018b) focused on testing the role of land use as a driver of biotic homogenization in bee communities in the northeastern United States. Both studies used the same data, which consisted of two datasets collected in 2014 and 2015, respectively, at 36 sites (12 of each habitat type) distributed across New Jersey, southern New York, and eastern Pennsylvania. Here, we again use data from all 36 of these sites. On each sampling occasion, blue vane traps and 3.25-ounce pan traps were deployed simultaneously for a 24-hour period during which weather was predicted to be favorable for bee activity (sunny to partly cloudy, with low chance of rain and high temperatures greater than 18°C). To minimize variation in trap effectiveness driven by environmental variables (e.g., floral abundance), traps were deployed at four locations within each site, each of which was in an area of mowed grass with minimal floral resources. At each location, pan traps were arranged in a line of six traps spaced at 1.5-meter intervals in alternating colors (blue, yellow, and white). At each of two haphazardly selected locations within each site, a single blue vane trap was also placed in the trap array. All traps were filled with water mixed with dish detergent.

Smith et al. 2021 — pan trap and vane trap:

The goal of this study was to measure how abundance and richness of forest-associated bee communities changed across forest fragments of different areas and ages. While data were collected in both 2017 and 2018, here we used only the 2018 dataset that was gathered using paired pan trap and vane trap sampling. This dataset was collected across 27 sites in central New Jersey. Sampling occurred only on days when temperatures exceeded 17°C. On each sampling occasion, 3.25-ounce pan traps and blue vane traps were deployed simultaneously for about 8 hours spanning peak midday bee activity. Pan traps were arranged in a 40 by 100-meter grid of 39 traps, divided evenly among three colors (blue, yellow, and white). Additionally, a blue vane trap was deployed each of the four corners of this grid. All traps were filled with water mixed with dish detergent.

Harrison et al. unpublished — pan trap and hand net:

This dataset, published here for the first time, was gathered as part of a study that aimed to collect bees from under-sampled and uncommon habitat types in New Jersey to better characterize the regional species pool and evaluate species rarity. Data were collected in 2016 at 32 sites across the state which were selected because 1) the 1-kilometer radius area around them was dominated by a single land cover type (agriculture, forest, urban, wetland, or water/beach) and 2) they were not within 10 kilometers of any site previously sampled by our lab group. Habitats that are unique to different geographic areas of the state (e.g., deciduous forest fragments in northern NJ, bogs and similar wetlands in southern NJ, beach dunes along the coast) were prioritized when selecting sites, as were field crops (e.g., soy, corn, potatoes, and wheat) that represented an under-sampled subset of agricultural habitats.

All sites were sampled twice in 2016 — once between April 1 and June 15 and again between June 16 and August 15. During each sampling occasion at a given site, 3.25-ounce pan traps were set up by 8:00 AM in a single transect of 35 traps spaced 1.5 meters apart and taken down at 3:00 PM the same day. While pan traps were deployed, a single observer also collected bees by hand net in four or five 30-minute rounds along transects radiating up to 50 meters outward from the pan trap transect (a circular sampling area with an area of 0.785 hectares). Observers intentionally avoided netting bumble bees (*Bombus* spp.), since most species of this genus that occur in New Jersey are relatively well-sampled and avoiding them would also minimize impacts of collecting on their populations.

**References**

Harrison T, Gibbs J, Winfree R. Forest bees are replaced in agricultural and urban landscapes by native species with different phenologies and life-history traits. Global Change Biology. 2018a; 24(1):287–96. https://doi.org/10.1111/gcb.13921

Harrison T, Gibbs J, Winfree R. Phylogenetic homogenization of bee communities across ecoregions. Global Ecology and Biogeography. 2018b; 27(12):1457–66. https://doi.org/10.1111/geb.12822

Smith C, Harrison T, Gardner J, Winfree R. Forest-associated bee species persist amid forest loss and regrowth in eastern North America. Biological Conservation. 2021; 260:109202. https://doi.org/10.1016/j.biocon.2021.109202

Winfree R, Williams NM, Dushoff J, Kremen C. Species Abundance, Not Diet Breadth, Drives the Persistence of the Most Linked Pollinators as Plant-Pollinator Networks Disassemble. The American Naturalist. 2014; 183(5):600–11. https://doi.org/10.1086/675716

**Methods S2. Resources used for species-level identification of bee specimens.**

Bouseman JK, LaBerge WE. A revision of the bees of the genus Andrena of the Western Hemisphere. Part IX. Subgenus Melandrena. Transactions of the American Entomological Society. 1978; 104(3/4): 275–389.

Gibbs J .Revision of the metallic Lasioglossum (Dialictus) of eastern North America (Hymenoptera: Halictidae: Halictini). Zootaxa. 2011; 3073(1): 1–216.

LaBerge WE. A revision of the bee genus Andrena of the Western Hemisphere. Part II. Plastandrena, Aporandrena, Charitandrena. Transactions of the American Entomological Society. 1969; 95(1): 1–47.

LaBerge WE. A revision of the bees of the genus Andrena of the Western Hemisphere. Part VI. Subgenus Trachandrena. Transactions of the American Entomological Society. 1973; 99(3): 235–371.

LaBerge WE. A revision of the bees of the genus Andrena of the Western Hemisphere. Part VIII. Subgenera Thysandrena, Dasyandrena, Psammandrena, Euandrena, Oxyandrena. Transactions of the American Entomological Society. 1977; 103(1): 1–143.

LaBerge WE. A revision of the bees of the genus Andrena of the Western Hemisphere. Part X. Subgenus Andrena. Transactions of the American Entomological Society. 1980; 106(4): 195–525.

LaBerge WE. A revision of the bees of the genus Andrena of the Western Hemisphere. Part XI. Minor subgenera and subgeneric key. Transactions of the American Entomological Society. 1985; 111(4): 441–567.

LaBerge WE. A revision of the bees of the genus Andrena of the Western Hemisphere. Part XII. Subgenera Leucandrena, Ptilandrena, Scoliandrena and Melandrena. Transactions of the American Entomological Society. 1986a; 112(3): 191–248.

LaBerge WE. A revision of the bees of the genus Andrena of the Western Hemisphere. Part XIII. Subgenera Simandrena and Taeniandrena. Transactions of the American Entomological Society. 1989; 115(1): 1–56.

LaBerge WE, Bouseman JK. A revision of the bees of the genus Andrena of the Western Hemisphere. Part III. Tylandrena. Transactions of the American Entomological Society. 1970; 96(4): 543–605.

LaBerge WE, Ribble DW. A revision of the bees of the genus Andrena of the Western Hemisphere. Part V. Gonandrena, Geissandrena, Parandrena, Pelicandrena. Transactions of the American Entomological Society. 1972; 98(3): 271–358.

LaBerge WE, Ribble DW. A revision of the bees of the genus Andrena of the Western Hemisphere. Part VII. Subgenus Euandrena. Transactions of the American Entomological Society. 1975; 101(3): 371–466.

McGinley RJ. Studies of Halictinae (Apoidea: Halictidae), I: Revision of New World Lasioglossum Curtis. Smithsonian Contributions to Zoology. 1986; 429: 1–294.

Mitchell, TB. Bees of the Eastern United States: volume I. North Carolina Agricultural Experimental Station Technical Bulletin. 1960; 141, 1–538.

Mitchell, TB. Bees of the Eastern United States: volume II. North Carolina Agricultural Experimental Station Technical Bulletin. 1962; 152, 1–557.

Rehan SR, Sheffield CS. Morphological and molecular delineation of a new species in the *Ceratina dupla* species-group (Hymenoptera: Apidae: Xylocopinae) of eastern North America. Zootaxa. 2011; 2873: 35–50.

Ribble DW. Revisions of two subgenera of Andrena: Micrandrena Ashmead and Derandrena new subgenus (Hymenoptera: Apoidea). Bulletin of the University of Nebraska State Museum. 1968; 8(5): 237–394.

Ribble DW. A revision of the bees of the genus Andrena of the western hemisphere subgenus Scaphandrena. Transactions of the American Entomological Society. 1974; 100(2): 101–189.

Sandhouse GA. The North American bees of the genus Osmia (Hymenoptera: Apoidea). Memoirs of the Entomological Society of Washington. 1939; 1: 1–167.

Stephen WP. A revision of the bee genus Colletes in America north of Mexico (Hymenoptera, Colletidae). The University of Kansas Science Bulletin. 1954; 36(6): 149–527.

Williams P, Thorp R, Richardson L, Colla S. Bumble Bees of North America. Princeton University Press (Princeton). 2014; 1–208.

**Table S1. Sampling effort by collection method across studies.** Studies collected bees using pan traps paired with either hand netting (yellow) or vane traps (blue). Effort for hand-netting is measured as the total number of hours spent sampling summed across all individual collectors (“collector-hours”). Effort for pan and vane traps is measured as the total number of hours of sampling summed across the total number of traps used in each sampling event (“trap-hours”).

| Study | Year | Collection Method | Effort | Sample Size |
| --- | --- | --- | --- | --- |
| Winfree et al. 2014 | 2006 | hand netting | 64 collector-hours | 578 |
|  |  | pan trap | 7,488 trap-hours | 2,357 |
| Harrison et al. unpublished | 2016 | hand netting | 152 collector-hours | 4,834 |
|  |  | pan trap | 18,620 trap-hours | 4,827 |
| Harrison et al. 2018a, Harrison et al. 2018b | 2014 | vane trap | 5,040 trap-hours | 272 |
|  |  | pan trap | 60,480 trap-hours | 4,121 |
| Harrison et al. 2018a, Harrison et al. 2018b | 2015 | vane trap | 6,912 trap-hours | 765 |
|  |  | pan trap | 82,944 trap-hours | 6,760 |
| Smith et al. 2021 | 2018 | vane trap | 1,728 trap-hours | 220 |
|  |  | pan trap | 16,848 trap-hours | 2,751 |

**Table S2. Taxonomic groupings used in species-level analyses.** Bee specimens belonging to four species complexes with unresolved taxonomy could not be identified with certainty below the species complex level and were grouped as such for species-level analyses in this study.

| **Species** | **Species Complex** |
| --- | --- |
| *Hylaeus affinis* | *Hylaeus affinis/modestus* |
| *Hylaeus modestus* | *Hylaeus affinis/modestus* |
| *Lasioglossum hitchensi* | *Lasioglossum hitchensi/weemsi* |
| *Lasioglossum weemsi* | *Lasioglossum hitchensi/weemsi* |
| *Nomada bella* | bidentate *Nomada* |
| *Nomada cuneata* | bidentate *Nomada* |
| *Nomada illinoensis* | *Nomada illinoensis/sayi* |
| *Nomada lepida* | bidentate *Nomada* |
| *Nomada ovata* | bidentate *Nomada* |
| *Nomada sayi* | *Nomada illinoensis/sayi* |

**Table S3. Focal genera and body size classes for comparison of methods’ relative biases.** Focal bee genera in each methodological comparison (pan trap vs. hand net and pan trap vs. vane trap) were those that were captured by at least one method on at least ten site dates.

| **Genus** | **Body Size Class** | **Comparison** | |
| --- | --- | --- | --- |
|  |  | *Pan trap vs. hand net* | *Pan trap vs. vane trap* |
| *Agapostemon* | medium | X | X |
| *Andrena* | medium | X | X |
| *Anthidium* | large | X | X |
| *Anthophora* | very large |  | X |
| *Augochlora* | small | X | X |
| *Augochlorella* | small | X | X |
| *Augochloropsis* | medium | X |  |
| *Bombus* | very large | X | X |
| *Calliopsis* | small | X | X |
| *Ceratina* | small | X | X |
| *Colletes* | large | X | X |
| *Eucera* | very large |  | X |
| *Halictus* | medium | X | X |
| *Heriades* | small | X |  |
| *Hoplitis* | small | X | X |
| *Hylaeus* | small | X | X |
| *Lasioglossum* | small | X | X |
| *Megachile* | large | X | X |
| *Melissodes* | large | X | X |
| *Nomada* | small | X | X |
| *Osmia* | medium | X | X |
| *Sphecodes* | small | X | X |
| *Xylocopa* | very large | X |  |

**Table S4. Summary of generalized linear mixed model results for evaluation of bee genus sampling bias.** Pan trap is fixed as the reference level in both models. The intercept is therefore a log-scale estimate of average genus-level bee abundance per sampling event (site-date combination) in pan traps, while the parameter estimate for the sampling method fixed effect expresses the (log scale) difference in average genus-level bee abundance between pan traps and either hand net or vane trap samples.

|  | **Intercept** | | **Fixed effect (method)** | | | **Random effect standard dev.** | | |
| --- | --- | --- | --- | --- | --- | --- | --- | --- |
| **Comparison** | **Estimate** | **SE** | **Estimate** | **SE** | **p-value** | **Sampling event (intercept)** | **Genus (intercept)** | **Genus (slope)** |
| pan trap vs. hand net | -1.09 | 0.50 | 0.40 | 0.34 | 0.24 | 1.10 | 2.24 | 1.49 |
| pan trap vs. vane trap | -0.69 | 0.37 | -2.13 | 0.37 | <0.001 | 0.87 | 1.61 | 1.59 |

**Table S5. Summary of generalized linear mixed model results for evaluation of bee size sampling bias.** Pan trap is fixed as the reference level in both models. The intercept is therefore a log-scale estimate of average genus size class bee abundance per sampling event (site-date combination) in pan traps, while the parameter estimate for the sampling method fixed effect expresses the (log scale) difference in average size class bee abundance between pan traps and either hand net or vane trap samples.

|  | **Intercept** | | **Fixed effect (method)** | | | **Random effect standard dev.** | | |
| --- | --- | --- | --- | --- | --- | --- | --- | --- |
| **Comparison** | **Estimate** | **SE** | **Estimate** | **SE** | **p-value** | **Sampling event (intercept)** | **Size (intercept)** | **Size (slope)** |
| pan trap vs. hand net | -0.92 | 1.09 | 0.61 | 0.67 | 0.36 | 0.83 | 2.16 | 1.31 |
| pan trap vs. vane trap | -0.27 | 0.62 | -1.36 | 0.61 | 0.02 | 0.78 | 1.22 | 1.20 |


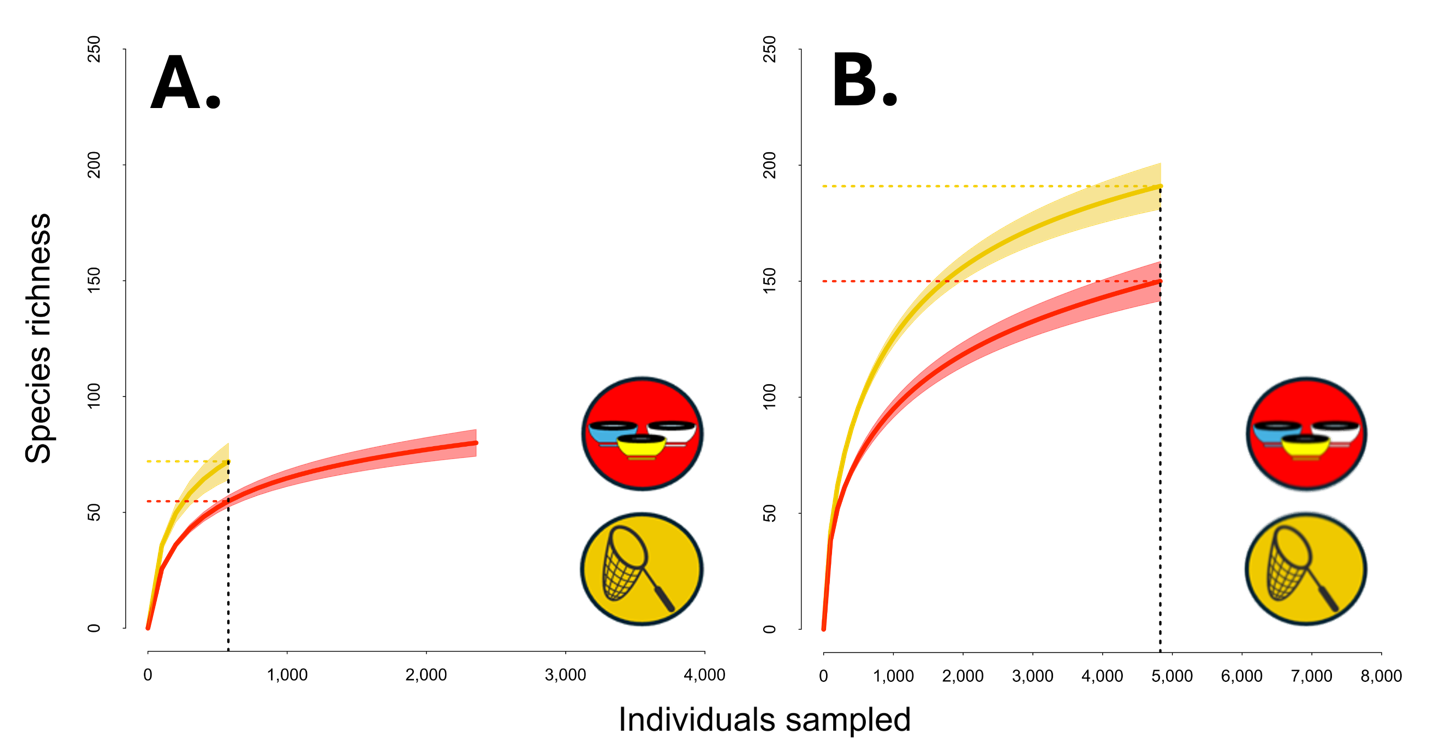


**Figure S1. Rarefaction curves and comparison of species richness for pan trap (red) and hand net (yellow) separated by individual study for a.) Winfree et al. 2016 and b.) Harrison et al. (unpublished).** Points show rarefied estimates of sample richness, with 95 percent confidence intervals. Vertical dashed lines (black) indicate the sample size for the method that captured the fewest individual bees, where we compare richness between each pair of methods; lack of overlap in 95 percent confidence intervals at this point is interpreted as a significant difference in richness between methods.


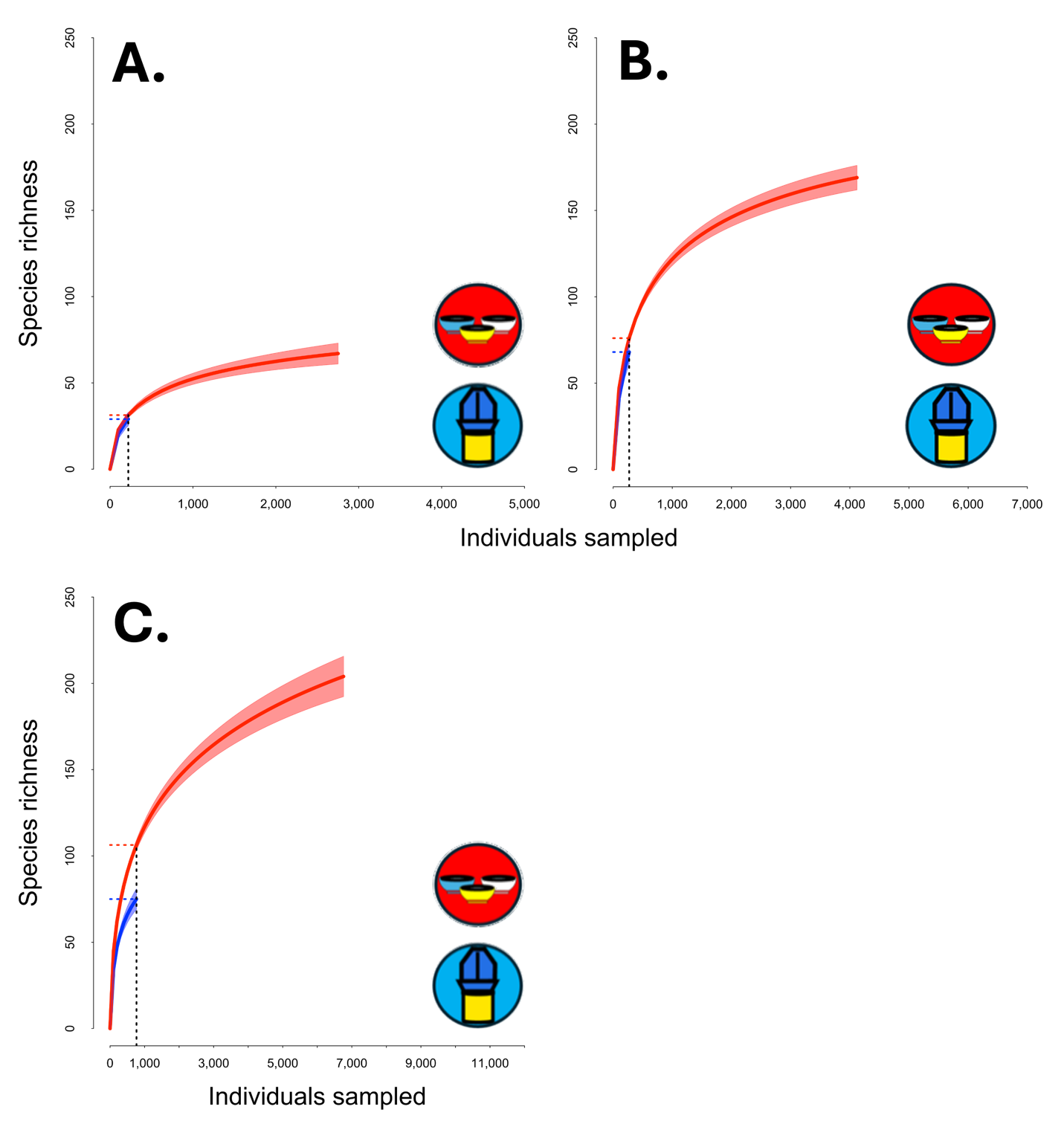


**Figure S2. Rarefaction curves and comparison of species richness for pan trap (red) and vane trap (blue) separated by individual study for a.) Smith et al. 2021 and b.) year 1 and c.) year 2 of Harrison et al. 2018** Points show rarefied estimates of sample richness, with 95 percent confidence intervals. Vertical dashed lines (black) indicate the sample size for the method that captured the fewest individual bees, where we compare richness between each pair of methods; lack of overlap in 95 percent confidence intervals at this point is interpreted as a significant difference in richness between methods.
